# Coagulation disorders during treatment with cefazolin and rifampicin: rare but dangerous

**DOI:** 10.5194/jbji-6-131-2021

**Published:** 2021-04-01

**Authors:** Ines Kouki, Clémence Montagner, Wladimir Mauhin, Jonathan London, Thierry Lazard, Sylvie Grimbert, Valérie Zeller, Olivier Lidove

**Affiliations:** 1 Service de Médecine Interne, Groupe Hospitalier Diaconesses – Croix Saint-Simon, 125 rue d'Avron, 75020, Paris, France; 2 Service de Réanimation, Groupe Hospitalier Diaconesses – Croix Saint-Simon, 125 rue d'Avron, 75020, Paris, France; 3 Service de Gastro-entérologie et Endoscopies Digestives, Groupe Hospitalier Diaconesses – Croix Saint-Simon, 125 rue d'Avron, 75020, Paris, France; 4 Centre de Référence des Infections Ostéo-articulaires, Groupe Hospitalier Diaconesses – Croix Saint-Simon, 125 rue d'Avron, 75020, Paris, France

## Abstract

We describe a 79-year-old man with spondylodiscitis and unknown pathogen, treated with cefazolin and rifampicin. He developed a massive digestive hemorrhage. Prothrombin time was prolonged with severe vitamin-K-dependent clotting-factor deficiency. Severe bleeding can occur during cefazolin and rifampicin use. This deficiency should be assessed before prescribing cefazolin–rifampicin and prothrombin time monitored.

## 1 Introduction

Cefazolin is a first-generation cephalosporin which is used more frequently to treat susceptible Gram-positive coccus infections (McDanel et al., 2017). Its rare adverse events include benign digestive disorders, *Clostridium difficile* colitis, neurological disorders at high dose, hepatic and renal manifestations, and hematological abnormalities with cytopenia and coagulopathies.

Rifampicin is a major antibiotic component of the treatment of tuberculosis and staphylococcal osteoarticular infections, especially of joint prosthesis. It always has to be combined with another antibiotic to limit the emergence of resistance. Rifampicin is a powerful enzymatic inducer of the cytochrome P450 system, responsible for drug interactions, necessitating adaptation of other treatments metabolized via the same pathway.

Cefazolin and rifampicin antibiotics can be combined, especially to treat staphylococcal osteoarticular infections. No drug interaction has been described with this combination, as cefazolin is excreted unchanged by the kidneys and only minimally in bile.

We report a patient with severe hypovitaminosis K, which was revealed during the use of this drug combination, and presented with necrotic esophagitis complicated by digestive hemorrhage.

## 2 Case presentation

A 79-year-old man was hospitalized for 6 weeks with L4–L5 spondylodiscitis in September 2019. His medical history consisted principally of membranoproliferative glomerulonephritis treated with rituximab several years ago (currently in remission), hypertension, alcoholic liver disease with chronic cholestasis without signs of portal hypertension, and elastometry fibrosis score estimated at F3. Hepatitis-C serology was negative. In August 2019, he developed sudden-onset low back pain with a marked inflammatory syndrome. Lumbar magnetic resonance imaging (Fig. S1 in the Supplement) showed what appeared to be infectious L4–L5 spondylodiscitis with a 17×12 mm infiltration in the adjacent right psoas, circumferential epiduritis and bilateral, but predominantly right, posterior L4–L5 articular arthritis. At this stage, renal function and a complete liver work-up were normal, with prothrombin time (PT) at 91 %. Factor V was not determined.

We performed two puncture disco-vertebral biopsies, but no pathogen could be isolated from them. *Brucella*, *Bartonella* and *Coxiella* serologies were negative. Assuming methicillin-sensitive *Staphylococcus* because of the radiographic appearance, or of non-hemolytic streptococci because of the poor oral hygiene of the patient, cefazolin was started at 2 g every 8 h, after the sampling of the different biopsies. However, histological examination of the two disco-vertebral biopsies found a granulomatous appearance with necrotic micro-foci, which led to the priority search for a mycobacterium, notably tuberculosis. His neurological deterioration led to emergency laminectomy on hospitalization day 5. Cultures of intra-operative samples remained sterile after prolonged incubation in enriched media and mycobacterial cultures. Histology of intra-operative samples showed necrotic micro-foci as well.

Because the diagnosis of infectious spondylodiscitis was certain, because of the negativity of standard and prolonged cultures, because of the presence of features compatible with both types of infections (classical bacteria and mycobacteria), and because of a high risk of complications, a multidisciplinary discussion (between internists and specialists in infectious diseases) led to cefazolin continuation for a total of 6 weeks combined with anti-tuberculous quadritherapy (isoniazid, rifampicin, ethambutol and pyrazinamide) for 2 months, followed by a dual therapy for a further 4 months.

On day 24 of cefazolin administration and day 7 of quadritherapy, the patient developed abundant hematemesis. Cefazolin was temporarily withdrawn. The laboratory work-up found PT at 5 %, normal factor V (76 %) and factors II, VII and X severely collapsed (respectively, 28 %, 2 % and 7 %). After intravenous (IV) vitamin-K (10 mg) supplementation and human pro-coagulation complex, endoscopy identified necrotic esophagitis of the lower two-thirds of the esophagus and a necrotic bulbar ulcer. Proton-pump inhibitors administered by electric syringe achieved rapid improvement; the control endoscopy 48 h later found the peptic esophagitis appearance markedly attenuated. No carcinomatous- or lymphomatous-like cellular infiltration was seen during histological examination of esophagus samples.

Determination of serum cefazolin concentrations did not indicate an overdose (continuous concentration of 33.4 mg/L for a targeted value of 20–40 mg/L). After vitamin-K supplementation, cefazolin was restarted for a total duration of 6 weeks, with close monitoring of hemostasis. The anti-tuberculous regimen reintroduced combined ethambutol, moxifloxacin, isoniazid and pyrazinamide.

## 3 Discussion

Cefazolin is a first-generation cephalosporin used to treat methicillin-sensitive *Staphylococcus aureus* infections. Only a parenteral formulation is available. Its therapeutic utilization is broad and adverse events are rare (Zeller et al., 2009). Its tolerance is better than that observed for oxacillin. Among described cefazolin adverse events are coagulation disorders associated with hypovitaminosis K. The latter is a rare side effect, occurring predominantly on a background at risk, such as malnutrition, pre-existing vitamin-K deficiency, neoplasia, renal insufficiency, recent surgery, or ileus (Chung and Watson, 2008). Those anomalies contribute to hemostasis disorders. They are mostly observed with certain cephalosporins (cefamandole, cefoperazone and moxalactam), whose nucleus carries a 1-methyltetrazole-5-thiol (MTT) group (Lipsky, 1983).

Attachment of vitamin-K-dependent factors to platelets is assured by their amino-terminal domain, which undergoes post-translational modification, i.e., gamma-carboxylation of glutamate residues by gamma glutamyl carboxylase. The MTT group interferes, in vitro, in vitamin-K metabolism by blocking vitamin-K–epoxide reductase, which intervenes in this carboxylation, and thus leads to five coagulation-cascade proenzymes (factors VII, IX, X, prothrombin and protein C) losing their ability to bind to the blood platelet surface (Dahlbäck, 2000; Sadler, 2004). Direct inhibition of gamma glutamyl carboxylase is possible too (Angles et al., 2018).

Cefazolin has a 2-methyl-1,2,3-thiadiazol-5-thiol (MTD) group – not an MTT group (Wood et al., 2002) – which seems to have the same properties as vitamin-K antagonists (Angles et al., 2018). These effects would be enhanced in a context of renal insufficiency and when high doses are administered, leading to the accumulation of this active metabolite. This heightened risk is now described in the VIDAL (https://www.vidal.fr/medicaments/cefazoline-panpharma-1-g-5-ml-pdre-sol-p-us-parenter-iv-3348.html, last access: 10 February 2021) (French Physician's Desk Reference). Some authors recommend systematic vitamin-K adjunction for certain cefazolin-treated patients.

Hypovitaminosis K has also been described with rifampicin alone (Sampaziotis and Griffiths, 2012). The origin of these coagulation disorders is multifactorial, attributable to a direct effect on the intestinal flora that participate in the production of vitamin K, inhibition of vitamin-K–epoxide reductase and acceleration of the degradation of vitamin K linked to hepatic enzyme induction.

Little is known about hemostasis disorders under cefazolin, but they seem to be rare. In 2016 and 2017, several cases were reported and notified to pharmacovigilance authorities. Indeed, when the stock of penicillin M was exhausted in 2016, learned societies in France recommended cefazolin as an alternative. PT collapse in a patient with active bleeding was described 7 d after its use alone to treat infectious endocarditis (Gay et al., 2018). In addition, four cefazolin–rifampicin-treated patients developed severe hemorrhaging with low PT; two of them died (Angles et al., 2018). As well, necrotic esophagitis was seen on day 5 of IV cefazolin administered for osteomyelitis with collapsed PT and vitamin-K-dependent factors (Barnes et al., 2014).

A retrospective French study conducted in 2018 evaluated the hemorrhagic risk of cefazolin (Strazzulla et al., 2019). Among the 132 patients included, 59 had available coagulation parameters. Activated partial thromboplastin time was significantly prolonged at the end of treatment for these 59 patients; 7 of them experienced a serious hemorrhagic event. However, their prolonged activated partial thromboplastin times and bleeding events did not coincide. The excess risk and preventive measures to be implemented were the object of a national alert and are now indicated in good practice recommendations.

Our patient had several risk factors for coagulation disorders under cefazolin–rifampicin: malnutrition with albumin at 22 g/L, recent surgery, prolonged hospitalization and alcoholic hepatopathy. A possible vitamin-K deficiency had not been sought, and thus no supplementation was given prior to the dual antibiotic regimen. Mechanisms of the coagulopathy observed in our patient are summed up in Fig. S2 in the Supplement.

In our department, specializing in the treatment of complex osteoarticular infections, we frequently prescribe cefazolin, combined or not with rifampicin, for prolonged durations (several weeks) and at high dose (60–80 mg/kg/24 h). Based on a retrospective series of 100 patients continuously infused with cefazolin for 42 d in our unit, combined with rifampicin for 29 patients, no serious hemorrhagic event was observed (Zeller et al., 2009). Over the last 20 years, this patient's major coagulation disorder was the only one observed in our unit. Compared to a recent publication (Angles et al., 2018) on patients treated for endocarditis, our patients were probably in better general and nutritional condition, rarely had chronic hepatopathies and were likely hospitalized for shorter durations. We now systematically monitor PT every week throughout the entire treatment duration.

## 4 Conclusion

Coagulation disorders can complicate cefazolin use, notably for patients with predisposing condition(s). Rifampicin seems to reinforce these anomalies. Our patient had several risk factors for hypovitaminosis K, which is a rare event. Hence, hemostasis parameters should be verified before starting this antibiotic and PT should be monitored during treatment to avoid serious hemorrhagic accidents. PT should be monitored in patients with risk factors such as hypoalbuminemia or significant liver disease.

## Supplement

10.5194/jbji-6-131-2021-supplement The supplement related to this article is available online at: https://doi.org/10.5194/jbji-6-131-2021-supplement.

## Data Availability

No data sets were used in this article.
