# Supplementary material for: Coagulation disorders during treatment with cefazolin and rifampicin: rare but dangerous
Source: J Bone Jt Infect. 2021 Apr 1;6(5):131–4. doi: 10.5194/jbji-6-131-2021 (PMC8131959; doi:10.5194/jbji-6-131-2021)
Supplement: The supplement related to this article is available online at: https://doi.org/10.5194/jbji-6-131-2021-supplement. [file jbji-6-131-supplement.zip › jbji-6-131-2021-supplement-title-page.pdf]

Supplement of J. Bone Joint Infect., 6, 131–134, 2021
https://doi.org/10.5194/jbji-6-131-2021-supplement
© Author(s) 2021. CC BY 4.0 License.

Supplement of

Coagulation disorders during treatment with cefazolin and rifampicin: rare but dangerous

Ines Kouki et al.

Correspondence to: Clémence Montagner (cmontagner@hopital-dcss.org)

- jbji-6-131-2021-supplement-title-page.pdf
- FigureS1.pdf
- FigureS2.pdf

The copyright of individual parts of the supplement might differ from the article licence.
