# Supplementary material for: Coagulation disorders during treatment with cefazolin and rifampicin: rare but dangerous
Source: J Bone Jt Infect. 2021 Apr 1;6(5):131–4. doi: 10.5194/jbji-6-131-2021 (PMC8131959; doi:10.5194/jbji-6-131-2021)

Figure S2 : Mechanisms of coagulopathy in our patient. Glu = glutamate. C° = carboxylation. Plt = platelet. Vit k = vitamine K. MTD = 2 methyl 1.2.3. thiadiazol 5 thiol group.

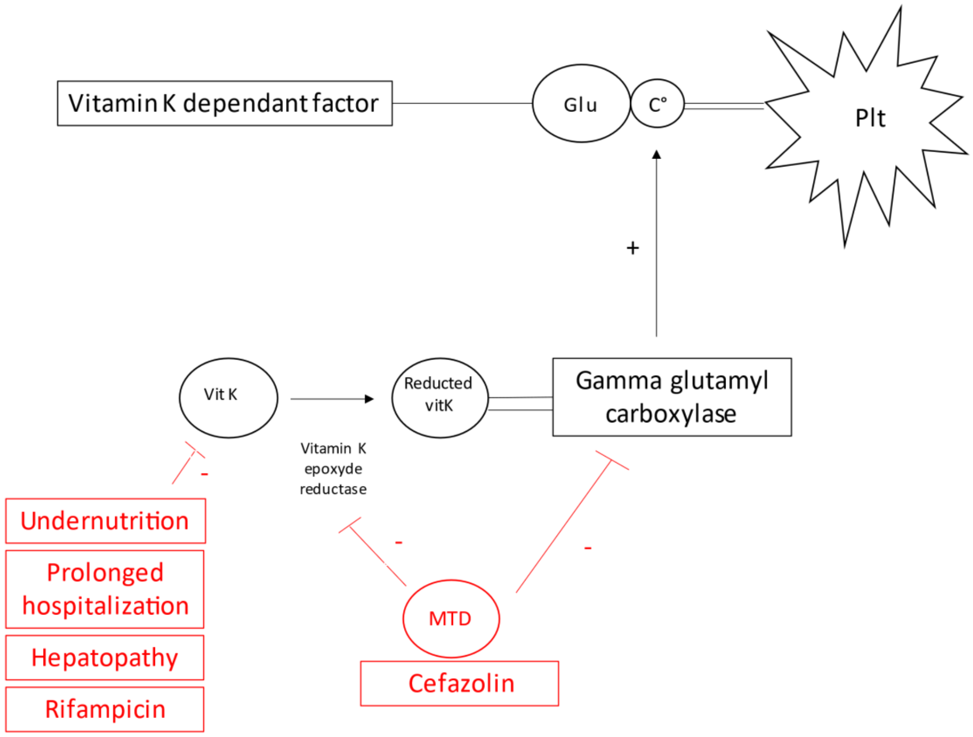

Supplement: The supplement related to this article is available online at: https://doi.org/10.5194/jbji-6-131-2021-supplement. [file jbji-6-131-supplement.zip › FigureS2.pdf]
