# Supplementary figures and images for: Coagulation disorders during treatment with cefazolin and rifampicin: rare but dangerous
Source: J Bone Jt Infect. 2021 Apr 1;6(5):131–4. doi: 10.5194/jbji-6-131-2021 (PMC8131959; doi:10.5194/jbji-6-131-2021)

Figure S1 : Lumbar Magnetic Resonance Imaging of our patient T1 Gadolinium 1

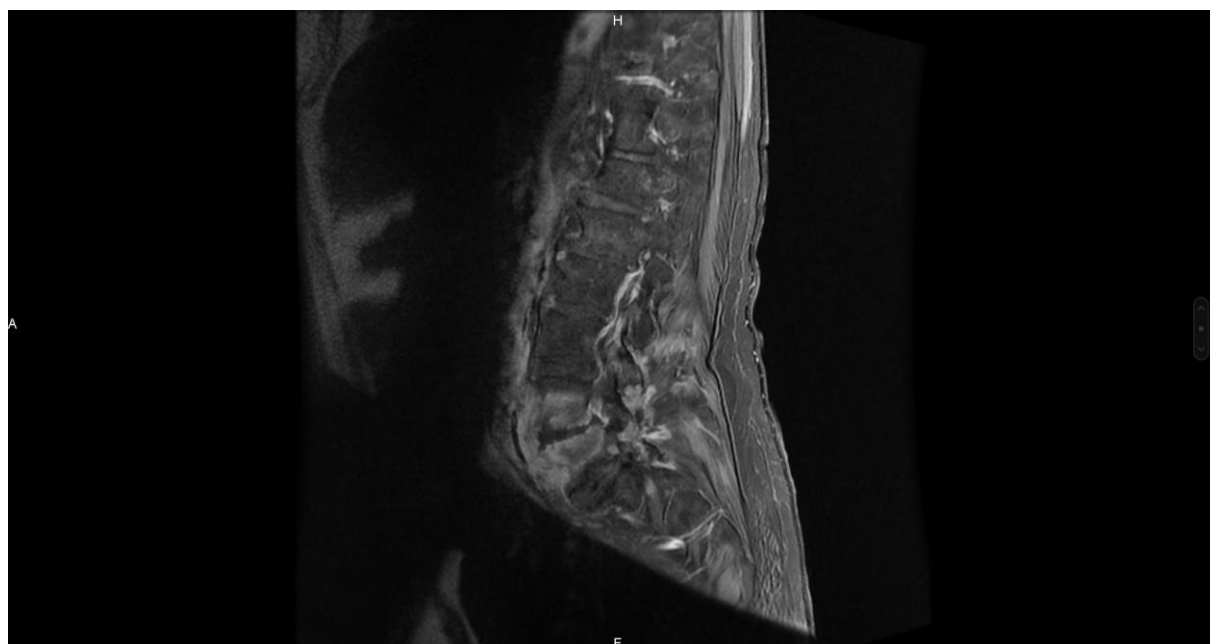

Supplement: The supplement related to this article is available online at: https://doi.org/10.5194/jbji-6-131-2021-supplement. [file jbji-6-131-supplement.zip › FigureS1.pdf]
